# Seizure Prophylaxis in Traumatic Brain Injury: A Comparative Study of Levetiracetam and Phenytoin Cerebrospinal Fluid Levels in Trauma Patients with Signs of Increased Intracranial Pressure Requiring Ventriculostomy

**DOI:** 10.7759/cureus.5784

**Published:** 2019-09-27

**Authors:** Bailey Zampella, Tye Patchana, James G Wiginton, James Brazdzionis, Marc Billings, Benjamin Archambeau, Alfonso Avila, Jeffrey Wang, Margaret Wacker, Dan E Miulli

**Affiliations:** 1 Neurosurgery, Riverside University Health System Medical Center, Moreno Valley, USA; 2 Emergency Medicine, Arrowhead Regional Medical Center, Colton, USA; 3 Pharmaceutical Sciences, Western University of Health Sciences, Pomona, USA; 4 Neurosurgery, Arrowhead Regional Medical Center, Colton, USA

**Keywords:** pharmacodynamics, keppra, levetiracetam, dilantin, phenytoin, traumatic brain injury, seizure, seizure prophylaxis, cerebrospinal fluid

## Abstract

Background

One of the most common life-threatening injuries to trauma patients arriving in the emergency department (ED) is traumatic brain injury (TBI). Traditionally, intravenous medications have been given as seizure prophylaxis in patients demonstrating signs of increased intracranial pressure (ICP), as post-traumatic seizures in trauma patients are associated with higher morbidity and mortality. Medications traditionally given for this indication such as phenytoin have been established to reach therapeutic levels in the cerebrospinal fluid (CSF) quickly and are effective in preventing post-traumatic seizures but often have a large side-effect profile. A newer medication that is being used for seizure prophylaxis in patients with epilepsy is levetiracetam. Levetiracetam typically has a better side effect profile, but it has not been demonstrated that the drug reaches therapeutic levels in the CSF as quickly as phenytoin. Studies have shown levetiracetam and phenytoin to be equivocal in the prevention of post-TBI seizure prophylaxis.

Methods

This was a prospective, randomized, case-control study at a Level II trauma center of adult patients (age >/= 18 years) who suffered severe TBI (sTBI) requiring the placement of an external ventricular drain (EVD) from May 2017 to June 2018. Twelve patients were randomly placed into one of two groups for the administration of antiepileptic medication (either levetiracetam or phenytoin), allowing for the subsequent serial collection of CSF for the analysis of therapeutic levels of antiepileptic medications. Levetiracetam or phenytoin was administered at standardized fixed doses per our neurosurgical center standard protocol. CSF was collected before either drug was administered, 60 minutes after completion of administration and 360 minutes after completion of drug administration. Data analysis was performed to compare the time frame for which therapeutic levels of the medications were achieved in the CSF. The published steady-state and therapeutic CSF level of levetiracetam is 32 mcg/ml and phenytoin is 2 mcg/ml.

Results

A trend was observed in which the closer the fixed dosage approximated the weight-based dosing of phenytoin, the more their CSF phenytoin level increased (and approximated the therapeutic range) with an associated R-squared value of 0.6274. This trend was not found in patients receiving levetiracetam.

Conclusions

Levetiracetam does not reach levels needed for seizure prophylaxis in human CSF when loaded at standard dosing regimens in the acute setting. Phenytoin does reach levels needed for seizure prophylaxis in human CSF with standardized regimen dosing when dosages approximate weight-based dosing. If needed, in the acute setting phenytoin should have additional doses given prior to six hours after the loading dose to achieve therapeutic CSF levels.

## Introduction

Traumatic brain injury (TBI) remains a major health problem in the United States, with more than one million injuries occurring per year, causing not only death but also long-term disability [1-2]. For patients who sustain a severe TBI (sTBI), as defined by a Glasgow Coma Score (GCS) of eight or less, antiepileptic therapy (AED) significantly decreases the incidence of early post-traumatic seizures [2-3]. Per our center's standard protocol, based on Brain Trauma Foundation guidelines, an external ventricular drain (EVD) is placed in patients who suffer sTBI, as defined by a GCS of eight or less [4]. In addition to monitoring and treating elevated intracranial pressures, the placement of an EVD allows for routine and regular monitoring of CSF constituents as surveillance. The administration of intravenous antiepileptic medications, such as levetiracetam or phenytoin, in the emergency department and/or intensive care unit (ICU), serve as prophylaxis in patients with sTBI who may develop increased intracranial pressure (ICP). Levetiracetam, a newer agent than phenytoin, has shown similar outcomes to decrease post-traumatic seizures [5]. Although medications traditionally given for this indication, such as phenytoin, have been established to reach therapeutic levels in cerebrospinal fluid (CSF) quickly and are effective in preventing early post-traumatic seizures, they often have a large side-effect profile [6]. Despite this, the prevention of post-traumatic seizures is critical, as post-traumatic seizures in sTBI trauma patients are associated with higher morbidity and mortality [6].

Per the landmark paper by Temkin, the use of phenytoin in sTBI shows a reduction in early post-traumatic seizures when compared to placebo [3]. An alternative medication that is being used for seizure prophylaxis in patients with sTBI is levetiracetam. Levetiracetam is not metabolized via the hepatic cytochrome p450 system, has a half-life of approximately six to eight hours, and two-thirds is excreted unaltered in the urine [5-6]. Levetiracetam has been found to have a lower side-effect profile, but currently, there are not significant evidentiary studies to demonstrate how long it takes the drug to reach therapeutic levels in the human CSF [7]. Currently, research is being conducted to investigate the dosing of levetiracetam (standard versus low dose, as well as in comparison to phenytoin) in regard to seizure prophylaxis after sTBI; however, further studies are needed [8]. The recent use of levetiracetam as seizure prophylaxis has increased due to the ease of dosing and a lack of need for drug level monitoring [5,9]. Multiple studies currently show similar outcomes based on the use of levetiracetam versus phenytoin for decreasing long-term post-traumatic seizure [5,9-11]. However, there is no current study that addresses the acute therapeutic level of levetiracetam in the CSF for effective seizure prophylaxis. The objective of our study is to acutely evaluate the CSF levels of levetiracetam versus phenytoin in patients with sTBI.

## Materials and methods

This was a prospective, non-blinded, randomized study conducted as a collaborative effort by the neurosurgery department and the emergency department at a level II trauma center from May 2017 to June 2018.

A total of 12 patients (six females, six males) were enrolled. Inclusion criteria included either male or female patients 18 years or older who presented with sTBI and required the placement of an EVD. Exclusion criteria included any patients under the age of 18 years old, patients with a known seizure disorder and who take antiepileptic medication regularly, patients with a known allergy to either levetiracetam or phenytoin, or those who were deemed to have a direct contraindication to either medication as established by the drug manufacturer. Patients were identified as having sTBI by a GCS of eight or less, requiring the placement of an EVD according to the guidelines for the management of severe head injury, and were randomly assigned to one of the two groups of the study. The levetiracetam group (six patients) were given only intravenous levetiracetam (standard dosing at our institution 1000 mg loading dose, followed by 1000 mg BID) and the phenytoin group (six patients) were given only intravenous phenytoin (standard dosing at our institution 1000 mg loading dose, followed by 200 mg BID typically scheduled at 0900/2100 first dose given at the next scheduled time).

Serial CSF samples were drawn at designated times, including prior to the administration of antiepileptic medication, which served as a baseline control, and then subsequently at one hour and six hours after the administration of antiepileptic medication. Samples were labeled with predetermined randomized numbers and stored in a -20 degree Celsius refrigerator and then was evaluated with high-performance liquid chromatography (HPLC).

The HPLC method for the simultaneous quantification of levetiracetam and phenytoin was developed using a Hewlett-Packard 1050 HPLC system (California, US), consisting of a degasser, a quaternary pump, and an autosampler. The system also included a diode array detector and a computer running Chemstation software (Rev. A.08.03; Agilent Technologies, California, US) for data acquisition and processing. Chromatographic separation was performed using a Waters Symmetry® C18 column (4.6 x 150 mm, 5 µm; Waters Corporation, Massachusetts, US). The analytes were quantified by ultraviolet (UV) detection at 220 nm. The mobile phase comprised water (A) and acetonitrile (B) and the flow rate was 1.0 ml/min. The elution began with 2% B followed by programmed elution: linear gradient to 25% B from zero to six min; linear gradient to 95% B from six to 12 min; linear gradient to 2% B from 12 to 13 min; and isocratic at 2% B from 13 to 15 min. The total run time was 15 min. Under these conditions, levetiracetam and phenytoin eluted from the column at 5.83 and 9.66 min, respectively. Samples were prepared using a protein precipitation procedure. Briefly, to a volume of 100 µl CSF sample in a microcentrifuge tube (1.7 ml) was added 10 µl 50% methanol, followed by 490 µl acetonitrile. The mixture was separated by centrifugation (140,000 rpm) at room temperature for 15 min. The supernatant (580 µl) was transferred to a borosilicate glass tube (12 x 75 mm) and evaporated to dryness with a stream of dry air. The residue was redissolved in 50% methanol (100 µl) and loaded into a plastic vial insert and 20 µl was injected into the HPLC system. Calibration curves were constructed from 0.5 to 20 µg/ml for both levetiracetam and phenytoin with good linearity (r > 0.99). Data analysis was completed using Microsoft Excel® (Microsoft Corporation, Washinton, US).

## Results

Twelve patients were included for analysis with HPLC. Of the 12, six patients received levetiracetam and six received phenytoin. HPLC results were tabulated to obtain CSF concentrations as listed in Tables 1-2. Goal concentrations were selected for 2 μg/mL for phenytoin and 12 μg/mL for levetiracetam [12-13]. Goal phenytoin and levetiracetam doses were calculated according to established norms of dosing for phenytoin and levetiracetam, respectively 17 mg/kg and 20 mg/kg [14]. Differences between goal CSF phenytoin and levetiracetam concentrations were calculated by subtracting the goal concentrations of CSF phenytoin and levetiracetam from the measured CSF phenytoin and levetiracetam measured by HPLC.

**Table 1:** Cerebrospinal fluid phenytoin concentration in μg/mL measured by high performance liquid chromatography Abbreviations: P: patient followed by patient number and sample number. Sample one as obtained prior to administration of antiepileptic, sample two was obtained one hour after administration of antiepileptic, and sample three was obtained six hours after administration of antiepileptic.

Patient Number	Calculated Cerebrospinal Fluid Phenytoin Concentration ( μg/mL)	Calculated Weight-Based Phenytoin Dose (mg)	Difference Between Calculated and Administered Phenytoin Dose (mg)	Difference Between Goal Phenytoin Concentration and Measured Concentration ( μg/mL)
P2-1	0.0	935	65	-2.0
P2-2	2.8	935	65	0.8
P2-3	2.3	935	65	0.3
P8-1	0.0	1853	-853	-2.0
P8-2	1.5	1853	-853	-0.5
P8-3	0.6	1853	-853	-1.4
P11-1	0.0	1309	-309	-2.0
P11-2	0.7	1309	-309	-1.3
P11-3	1.0	1309	-309	-1.0
P14-1	0.0	1207	-207	-2.0
P14-2	3.2	1207	-207	1.2
P14-3	2.5	1207	-207	0.5
P15-1	0.0	1326	-326	-2.0
P15-2	3.7	1326	-326	1.7
P15-3	1.4	1326	-326	-0.6
P21-1	0.0	1088	-88	-2.0
P21-2	1.0	1088	-88	-1.0
P21-3	1.5	1088	-88	-0.5

**Table 2:** Difference between measured concentration of levetiracetam in the CSF (𝞵g/mL) with goal concentration Abbreviations: P: patient followed by patient number and sample number. Sample one as obtained prior to administration of antiepileptic, sample two was obtained one hour after administration of antiepileptic, and sample three was obtained six hours after administration of antiepileptic.

Patient number	Calculated Levetiracetam Concentration (μg/mL)	Calculated Weight-Based Levetiracetam Dose (mg)	Difference Between Calculated and Administered Levetiracetam Dose (mg)	Difference Between Goal Levetiracetam Concentration and Measure Concentration (μg/mL)
P6-1	0.0	1860	-860	-12.0
P6-2	4.5	1860	-860	-7.5
P6-3	15.4	1860	-860	3.4
P10-1	0.1	1360	-360	-11.9
P10-2	17.0	1360	-360	5.0
P10-3	15.6	1360	-360	3.6
P12-1	0.1	1760	-760	-11.9
P12-2	7.8	1760	-760	-4.2
P12-3	7.8	1760	-760	-4.2
P13-1	0.2	1900	-900	-11.8
P13-2	7.9	1900	-900	-4.1
P13-3	7.6	1900	-900	-4.4
P18-1	0.0	1180	-180	-12.0
P18-2	11.2	1180	-180	-0.8
P18-3	6.6	1180	-180	-5.4
P20-1	0.0	1560	-560	-12.0
P20-2	13.3	1560	-560	1.3
P20-3	10.5	1560	-560	-1.5

**Figure 1:**
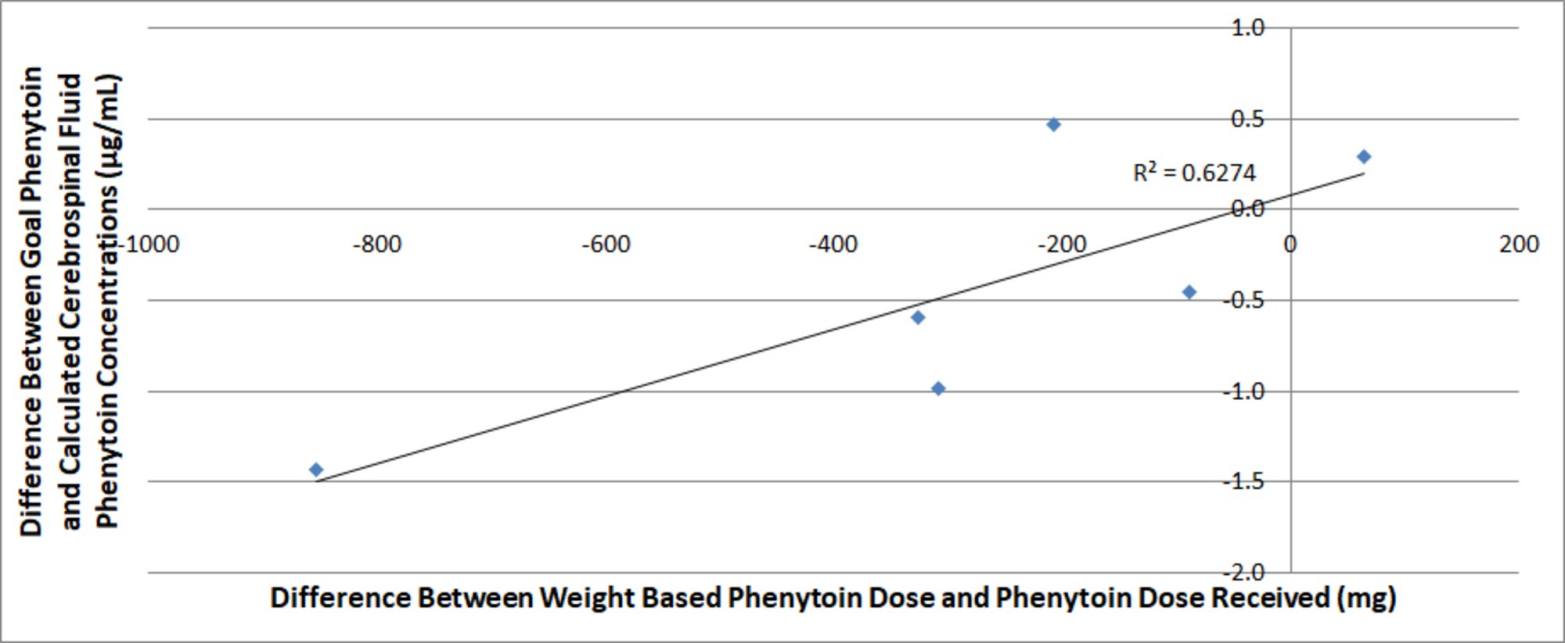
A comparison of weight-based phenytoin dosing compared to differences between goal and calculated cerebrospinal fluid phenytoin concentrations six hours after dosage

**Figure 2:**
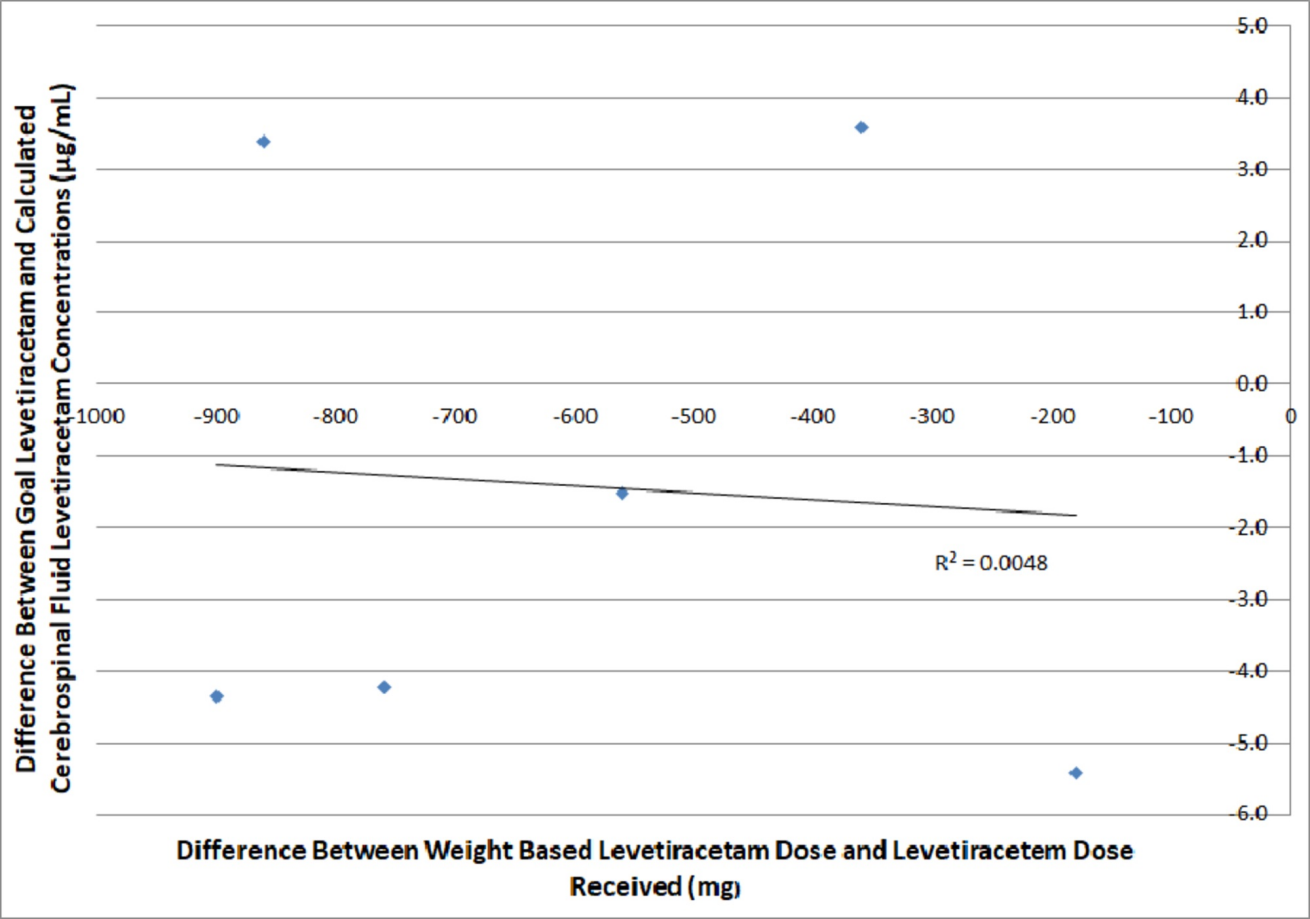
A comparison of weight-based levetiracetam dosing compared to differences between goal and calculated cerebrospinal fluid levetiracetam concentrations six hours after dosage

Differences between actual dose and weight-based dosing of phenytoin and levetiracetam compared to goal six-hour CSF concentrations of levetiracetam and phenytoin are plotted below in Figures 1-2. On the y-axis, positive values are those above minimum threshold CSF concentrations and negative values are those below minimum threshold CSF concentrations for phenytoin and levetiracetam, 2.0 μg/mL and 12.0 μg/mL, respectively. Further positive values on the x-axis represent doses of antiepileptic greater than goal dose while those with negative values represent the milligrams less than goal dose the patient received.

Figures 1-2 identify the difference between the administered and calculated dosing of antiepileptic and goal and calculated CSF concentrations of the antiepileptic. Figure 2 shows that there was not a predictable trend correlating variances or proximity of dosing to goal weight-based dose of levetiracetam to variances in the measured to goal CSF concentration. Figure 1 identifies a trend in which the closer the patient's dose was to the weight-based goal phenytoin, the more their CSF phenytoin level increased with an R-squared value of 0.6274.

## Discussion

The current literature demonstrates that levetiracetam, when recommended blood level doses are reached, can significantly increase the seizure-free time period following post-traumatic brain injury [15]. Recommended levetiracetam dosage ranges from 1000 - 4000 mg/day, with median dosage beginning at 3000 mg/day [13-15]. The therapeutic range of levetiracetam has not yet been definitively defined, however, a trough level between 12 and 46 μg/mL has been suggested in the literature [16-18].

A variety of laboratory methods have been utilized to measure levetiracetam levels in biological matrices, including but not limited to, immunoassays, HPLC with UV detection, gas chromatography with mass spectrometry detection, high-performance liquid chromatography-tandem mass spectrometry, capillary electrophoresis with UV detection, and ultra-performance liquid chromatography tandem-mass spectrometry [12]. Some of these methods require large sample volumes or lengthy run times to determine levetiracetam concentrations.

Our patient population consisted of two groups of six patients with sTBI who underwent EVD placement. For seizure prophylaxis, these two groups received levetiracetam and phenytoin, respectively. Studies have shown that seizures within the first-week post-traumatic brain injury can be decreased with AED administration [19]. Further studies have also explored the differences between levetiracetam and phenytoin as AEDs of choice [20]. Our study sought to evaluate the differences in CSF concentration between levetiracetam and phenytoin. We further sought to evaluate if standard dosage regimens were appropriate as compared to a weight-based dosage regime. With the placement of EVDs, we were able to draw CSF in order to evaluate the levels of AEDs present within the CSF. This was done via HPLC. Goal concentrations were selected as 2 μg/mL for phenytoin and 12 μg/mL for levetiracetam [12-13]. Calculated levels of phenytoin and levetiracetam were made, correcting from baseline measurements of CSF prior to AED administration. Also demonstrated were the difference between weight-based calculations and our standard dosing regimens used for seizure prophylaxis (Tables 1-2).

The current approach to antiepileptic prophylaxis involves the administration of either intravenous or oral medications to produce high levels of the medications in the bloodstream. Regardless of the administration, the medication must travel from the bloodstream to the brain tissue. The largest obstacle with the administration of such medications is the blood-brain barrier (BBB), which is composed of tight junctions between endothelial cells, with an absence of fenestrations and strict regulation of molecules from the blood into the brain interstitial fluid. Given this challenge, there are several new approaches to increase the entry and persistence of antiepileptic medications in the brain, including strategies such as drug delivery systems, prodrugs, efflux pump inhibition, hyperosmolar BBB opening, and circumventing the BBB via direct drug delivery to the ventricle and cortex [10].

Our study suggests that phenytoin reaches appropriate CSF concentration levels more predictably than those of levetiracetam when doses approximating weight-based calculations are used. As phenytoin levels approached weight-based dosing, CSF levels increased toward the therapeutic range. This relationship was not appreciated when plotting CSF levetiracetam levels against given and weight-based dosages (Figures 1-2). Although both groups did have loading doses less than the calculated weight-based doses, only the phenytoin group demonstrated predictability in measured CSF concentrations. Altogether, this suggests that appropriate CSF concentrations may be more easily achieved using phenytoin over levetiracetam intravenously.

Some of the limitations associated with this study have been identified. Data were collected from only one level II trauma center over a year-long period. We appreciate that, with just one center participating in the study, center-specific patient characteristics may contribute to the outcome. Furthermore, this study evaluated the CSF concentrations and pharmacodynamics of selected AED therapy but did not investigate the outcomes or incidence of post-traumatic seizure in this population. Further studies could be conducted to investigate this. Additionally, our study would benefit from larger sample sizes.

## Conclusions

The administration of AEDs are paramount to the prevention of seizure following TBI. Our study suggests that phenytoin reaches appropriate CSF concentration levels over that of levetiracetam when doses approximating weight-based calculations are used. Also, a correlation was found between the weight-based dosing of AED and higher concentration in CSF. More studies will be required in the future to determine the significance of this relationship.
